# Innovative Gluten-Free *Fusilli* Noodle Formulation: Leveraging Extruded Japanese Rice and Chickpea Flours

**DOI:** 10.3390/foods14142524

**Published:** 2025-07-18

**Authors:** Simone de Souza Fernandes, Jhony Willian Vargas-Solórzano, Carlos Wanderlei Piler Carvalho, José Luis Ramírez Ascheri

**Affiliations:** 1 Postgraduate Program in Food Science and Technology, Rural Federal University of Rio de Janeiro, Rodovia BR 465, km 7, Seropédica 23890-000, RJ, Brazil; simonesfernandes24@gmail.com; 2 Embrapa Agroindústria de Alimentos, Av. das Américas, 29501, Guaratiba 23020-470, RJ, Brazil; vargasjw@gmail.com (J.W.V.-S.); carlos.piler@embrapa.br (C.W.P.C.)

**Keywords:** gluten-free product, pasta-like product, extruded flour, extrusion cooking, physical property, chemical composition, cooking test

## Abstract

Background: The growing demand for nutritionally balanced, gluten-free products has encouraged the development of innovative formulations that deliver both sensory quality and functional benefits. Combining rice and legume flours offers promising alternatives to mimic gluten-like properties while improving nutritional value. This study aimed to develop a gluten-free fusilli noodle using extruded flours based on mixtures of Japanese rice (JR) and chickpea (CP) particles. Methods: A 2^3^ factorial design with augmented central points was applied to evaluate the effects of flour ratio (*X*_1_, CP/JR, 20–40%), feed moisture (*X*_2_, 24–30%), and extrusion temperature (*X*_3_, 80–120 °C) on responses from process properties (PPs), extruded flours (EFs), and noodle properties (NPs). Results: Interaction effects of *X*_3_ with *X*_1_ or *X*_2_ were observed on responses. On PP, *X*_1_ at 120 °C reduced the mechanical energy input (181.0 to 136.2 kJ/kg) and increased moisture retention (12.0 to 19.8%). On EF, *X*_1_ increased water-soluble solids (2.3 to 4.2 g/100 g, db) and decreased water absorption (8.6 to 5.7 g/g insoluble solids). On NP, *X*_1_ also affected their cooking properties. The mass increase was greater at 80°C (140 to 174%), and the soluble-solids loss was greater at 120 °C (9.3 to 4.5%). The optimal formulation (*X*_1_–*X*_2_–*X*_3_: 40–30%–80 °C) yielded noodles with improved elasticity, augmented protein, and enhanced textural integrity. Conclusions: Extruded flours derived from 40% chickpea flour addition and processed under mild conditions proved to be an effective strategy for enhancing both the nutritional and technological properties of rice-based noodles and supporting clean-label alternative products for gluten-intolerant and health-conscious consumers.

## 1. Introduction

Gluten-free noodles address the growing demand from consumers with celiac disease and gluten intolerance. In South America, Brazil leads this market segment, which is anticipated to expand as more consumers, such as those inclined to fitness and sports and others who associate it with enhanced digestion, are joining this marked [1]. To achieve high-quality gluten-free noodles, flours must exhibit excellent hydration capacity, elasticity, and stability, characteristics that depend on how their biopolymers—primarily from starches, proteins, and fibers—interact to form an elastic-cohesive network that gives the noodles their firmness.

Starch is the major component in flours from either cereals or pulses. When flours from these two sources are blended, the starch polymers are more readily available to react. In Japanese rice, the starch granules are almost entirely amylopectin, which gives it glutinous properties that aid in the formation of cohesive doughs [2,3,4]. On the other hand, in pulses, such as chickpeas, about one-third of the starch granules is amylose, which gives it structural properties that contribute to the firmness of the product. In addition, the proteins and fibers from the whole grains of chickpeas play an important role in retaining water and thus helping to plasticize the noodle dough [5].

The biopolymers in raw flours are compact entities that require slight modifications to improve the noodles’ firmness, and extrusion cooking is the ideal technology to carry out these modifications [6], which include surface fissures, stretching, melting, and/or breaking of starch granules, protein bodies, and fiber bundles. Depending on starch granule conversion degree and disentanglement or agglomeration of proteins, water absorption and water solubility of extruded flour components are affected. This influences the structuring of fresh doughs into a specific shape and maintaining that shape during the subsequent drying process or when cooking fresh or dry noodles to an ‘al dente’ texture, product characterized by being of firm hardness, elastic, and chewy [7].

Previous research used extruded flours based on cereal–legume mixtures for gluten-free noodle production, for enhancing its nutritional and technological characteristics. Rice was mainly used as base flour [8,9,10], which provides the necessary starch structure, while fiber and protein of pulse flours (yellow peas, chickpeas, and lentils) acts as a gluten substitute, significantly improving dough elasticity and nutritional quality [8,9]. This combination also increases darkness, reduces noodle expansion, brightness, and cooking losses, and positively influences the sensory acceptance of the final noodle product [8,10].

In addition, depending on the inclusion level of pulse flour and the cooking temperature for producing extruded flours, properties like the color, firmness, and adhesiveness can vary. From a health perspective, if high levels of resistant starch prevail in the final product, this may have beneficial effects on postprandial glycemic responses [8,9].

The moisture content used during extrusion, applied as a pre-cooking step to produce the gluten-free flour, significantly influences the resulting noodle texture, expansion, and cooking characteristics. Moisture levels of 24%, 27%, and 30% were selected based on previous studies, which showed that moisture below 24% results in brittle structure, while levels above 30% compromise dough cohesion and final texture [11].

Extrusion temperature is another critical parameter affecting gluten-free noodle quality, as it affects starch gelatinization and protein modification. The temperatures of 80 °C, 100 °C, and 120 °C were strategically selected to optimize these transformations and achieve optimal noodle structure and texture. Temperatures below 80 °C may inadequately gelatinize starch, while those above 120 °C risk degrading the product’s components and compromising overall quality [12].

Due to the thermo-mechanical process involved in extrusion, one approach to not over-transform natural biopolymers may be to feed coarse particles of material to the extruder [13]. In this work, coarse particles ≤ 1.40 mm of whole chickpea and polished rice (subsp. *japonica*) were blended as a study factor to complement their biopolymers, and it was assumed that coarse particles cooked by extrusion within suitable moisture and temperature ranges can generate extruded flours with improved technological properties for shaping in cold extrusion to produce gluten-free noodles with cooking and texture characteristics acceptable for consumption.

The objective of this study was to develop gluten-free noodles using extruded flours. The extrudates were obtained by varying three processing factors: flour ratio of mixtures based on chickpea (CP) and Japanese rice (JR) particles (CP/JR: 20/80-40/60, db), feed moisture (24–30%), and barrel temperature in the third heating zone (80–120 °C). The physical characterization in the extrusion and drying processes, in the extruded flours, and in theprepared noodles, was carried out to quantify the biopolymers’ modification degree in the feed material and its impact on cooking tests and instrumental texture of the noodles.

## 2. Materials and Methods

### 2.1. Production of Raw Flours

Short-grains of polished rice (Japanese variety, type (1)) were purchased from the Momiji brand (Camil Alimentos, São Paulo, SP, Brazil), whose production area is located in the south of Brazil (variety BRS 358). Chickpeas were kindly donated by Indústria de Alimentos Granfino^®^ (Nova Iguaçu, RJ, Brazil). To highlight the difference in pasta properties between the Japanese rice and a commercial rice, long-fine grains of polished rice (indica variety, type (2)) were purchased from the Palmares brand (Cooperativa Arrozeira Palmares, Palmares do Sul, RS, Brazil). The grains were milled into grits (≤1.400 mm) in two steps using an LM3600 disc mill (Perten Instruments AB, Huddinge, Sweden) with a gap between discs set at position #2. The milled products were manually sieved (Newark, NJ, USA), and coarse particles > 1.40 mm were passed through the disc mill again (position #0 for rice and #2 for chickpea). The resulting raw granulated ingredients were packaged in hermetically sealed plastic bags and stored under refrigeration (~7 °C) for a maximum of 7 days prior to use. Before preparing the mixtures, the moisture content of the raw Japanese rice ingredient (JR, 13.39 ± 0.05%) and chickpea ingredient (CP, 11.36 ± 0.01%) was determined. Then, CP/JR proportions were prepared at three levels: 20/80, 30/70, and 40/60 (dry basis). The moisture content of the 20/80 and 40/60 mixtures was adjusted to 24% and 30%, and the 30/70 mixture to 27%. The five mixtures were left to stand for 16 h at room temperature to equilibrate the moisture content. After this period, the moisture contents of them were verified: 20/80–24% (23.56 ± 0.05%), 40/60-24% (23.71 ± 0.17%), 20/80–30% (29.69 ± 0.07%), 40/60-30% (29.93 ± 0.01%), and 30/70–27% (26.02 ± 0.22%). The decrease in moisture content was less than 1%, which ensured compliance with the experimental design. The centesimal composition of the mixed flours was evaluated for moisture content, total proteins (nitrogen conversion factor for rice and products of 5.95), and ash [14]. The determination of total lipids was performed according to Bligh and Dyer [15]. Dietary fiber was quantified using Megazyme assay kids, and carbohydrate content was determined by difference.

### 2.2. Pasting Properties (RVA)

The paste viscosity properties of samples were evaluated using a Rapid Visco Analyzer model RVA-4 (Newport Scientific Pty Ltd., Warriewood, Australia), employing the Standard Analysis 1 profile. Analyses were conducted with 3 g of flour samples corrected to 14% moisture (wet basis).

The parameters obtained included peak viscosity (*PV*), indicating the maximum viscosity during heating; gelation viscosity (*GV*), the maximum viscosity during the cold holding phase; trough viscosity (*TV*), the minimum viscosity between *PV* and *GV*; breakdown viscosity (*BV* = *PV* − *TV*), representing the stability of swollen granules under heat and shear; and setback viscosity (*SV* = *GV* − *TV*), reflecting the tendency of starch molecules to retrograde during cooling.

### 2.3. Production of Extruded Flours

Extruded flours were produced following a 2^3^ factorial experimental design with four augmented central points. The studied factors and their actual levels are detailed in Table 1. Factor *X*_1_ represents the proportion of chickpea flour (%) combined with Japanese rice. Factor *X*_2_ corresponds to the adjusted moisture content (%), and factor *X*_3_ refers to the barrel temperature in the third zone. The conditioned CP/JR mixtures (feed materials) were processed using a 19/20 DN single-screw extruder (Brabender, Duinsburg, Germany), wherein the motor shaft was attached to a torque rheometer PlastiCorder LabStation (Brabender, Duinsburg, Germany). The screw coupled to the motor shaft had a compression ratio 2:1 and rotated at 150 rpm. Extruder barrel temperatures were set at 50 and 80 °C, for feeding and compression zones, respectively. The extruder die was the third heating zone, which was heated according to the conditions described in Table 1. The nozzle hole at the extruder die exit had a diameter of 4 mm. Feed materials were fed to the extruder at 4 kg/h through a volumetric feeder (Brabender, Duisburg, Germany), wherein the feeder screw speed was varied from 8 to 19 rpm (Table 1) due to bulk density differences between them.

Freshly extruded samples, which showed no significant expansion, were immediately cut and dried at 60 °C in a fan-forced oven. The dried samples were then milled using an LM3600 disc mill (Perten Instruments AB, Huddinge, Sweden) with a gap between discs set at position #12. The resulting coarse particles (diameter < 4 mm) were fed to a Quadrumat Jr. roller mill (Brabender, Duisburg, Germany), and the resulting milled products were manually sieved through laboratory mesh sieves (Newark, NJ, USA). The sieved products composed of particles ≤ 0.500 mm were labeled as extruded flours (EFs), which were packaged in hermetically sealed plastic bags and stored under refrigeration (~7 °C) for up to three months. A schematic illustration of the process is provided as Supplementary material (Figure S1).

### 2.4. Physical Characterization in the Extrusion and Drying Processes

The mass flow rate ( $\dot{m}$, kg/h) was measured at the beginning and at the end of each treatment, and together with the recorded torque (*T*, N·m), that were used to determine the specific mechanical energy (*SME*, kJ/kg) delivered to the material being extruded (Equation (1)) [16]. The moisture content of the extrudates collected and after drying was determined by the AOAC method 925.09 [14].

(1) $$SME=\frac{T\times 2\pi \times N}{\left(\dot{m}\right)}$$

### 2.5. Physical and Chemical Characterization of Extruded Flours

The EFs were characterized by the technological properties: water absorption index (*WAI*, g water/g is) and water solubility index (*WSI*, g ss/100 g db) (Equations (2) and (3)) [17].

(2)
W
S
I
=
g
 
w
a
t
e
r
 
s
o
l
u
b
l
e
 
s
o
l
i
d
s
(
s
s
)
g
 
d
r
y
 
s
a
m
p
l
e(
d
b)
×
100

(3)
W
A
I
=
g
 
a
b
s
o
r
b
e
d
 
w
a
t
e
r
g
 
d
r
y
 
s
a
m
p
l
e
×
(
1
−
s
o
l
u
b
l
e
 
f
r
a
c
t
i
o
n
)
=
g
 
a
b
s
o
r
b
e
d
 
w
a
t
e
r
g
 
i
n
s
o
l
u
b
l
e
 
s
o
l
i
d
s
(
i
s
)

The instrumental color of the EFs was determined using a colorimeter (Konica Minolta, CR-5, Tokyo, Japan). The color parameters were according to the CIELAB scale: chromaticity coordinates (a* and b*) and lightness (L*). The chemical composition of the extruded flours was determined following the same procedures used for the raw ingredients.

### 2.6. Noodle Production

The ingredients for the noodles were prepared using 300 g of EF. The flour (100%) was first mixed with salt (1%) in an N50A planetary mixer (Hobart Corp, North York, ON, Canada) for 3 min. Water (36%) was then gradually added over 7 min, based on the water absorption characteristics of each treatment. The kneading process lasted approximately 20 min. The dough was then shaped into fusilli-type noodles using a pasta extruder (Pastaia 2, ITALVISA, Tatuí, SP, Brazil). After shaping, the noodles were dried in an SL102 tray dryer (SOLAB, São Paulo, SP, Brazil), cooled to room temperature, packed in airtight plastic bags, and stored under refrigeration (~7 °C) for up to three months. A schematic illustration of the process is provided as Supplementary material (Figure S1).

### 2.7. Noodle Cooking Tests

Cooking tests according to the AACC method 16–50 [18] consisted of cooking 10 g of noodle in 140 mL of boiling distilled water. Every 15 s after 5 min of cooking, a sample was taken to check for the presence of raw material in the central axis. The optimum cooking time (*OCT*) of a treatment was established when no visual traces of raw material (total starch gelatinization) was confirmed. Once the *OCT* was found, the mass increase (*MI*) after draining of cooked noodles was calculated (Equation (4)):

(4) $$MI=\frac{mass\;of\;cooked\;sample}{mass\;of\;uncooked\;sample}\times 100\%$$

The volume of the raw and cooked noodles (at the *OCT*) was determined by water displacement. The mass of the set was recorded in a 200 mL graduated cylinder filled with water up to the top. After removing half of the water, the sample was inserted, completing the volume with the remaining water up to the top. The displaced volume corresponds to the volume of the sample. The volume increase after draining of cooked noodles was calculated (Equation (5)):

(5) $$VI=\frac{Volume\;of\;cooked\;sample}{Raw\;sample\;volume}\times 100\%$$

The soluble solids loss ( SSL) was determined by evaporation in an oven at 105 °C of 25 mL of the cooking water until constant mass was obtained (Equation (6)). Wheat noodles samples were used for comparison purposes.

(6) $$SSL=\frac{mass\;of\;evaporated\;residue}{mass\;of\;uncooked\;sample}\left(\frac{140\;mL}{25\;mL}\right)\times 100\%$$

### 2.8. Texture Profile Analysis of Noodles

The cooked noodles at the *OCT* were subjected to two compression cycles with a TA-XT Plus texture analyzer (Stable Micro System, Surrey, UK) equipped with a load cell of 5 kg. The cylindrical probe, with a base of 50 mm in diameter, descended at 1 mm/s until deforming the sample by 50% of its initial height, with an interval of 5 s between cycles. The force curves were recorded by the Exponent software version 6.1.11.0. The texture parameters extracted from the force profiles were hardness (*HA*), elasticity (*EL*), cohesiveness (*CO*), gumminess (*GO*), and chewiness (*CW*).

### 2.9. Statistical Analysis

Data obtained according to the $2^{3}$ factorial geometry with four augmented central points can generate predictive response surfaces (Equation (7)):

(7) $$Y=\beta_{0}+\beta_{1}x_{1}+\beta_{2}x_{2}+\beta_{3}x_{3}+\beta_{12}x_{1}x_{2}+\beta_{13}x_{1}x_{3}+\beta_{23}x_{2}x_{3}+\epsilon$$

where *Y* is the response evaluated in the following properties: (1) during the extrusion-drying process, (2) of the extruded flour, or (3) of the noodle formulated with extruded flour. *x_i_* represents the main effect of a study factor at coded levels, *x_i_ x_j_* represents the interaction effect between two study factors, and *β_i_* and *β_ij_* represent, respectively, linear and interaction coefficients of the model, which will be obtained by regression analysis. This analysis aims to minimize the experimental error of the model (*ϵ*), and for this purpose the Statistica software (StatSoft, Tulsa, OK, USA) was used.

Predictive models were generated for equations with R^2^ > 0.70 and nonsignificant lack of fit, which were presented as response surfaces. In the generated response surfaces, the constant factor level was set to its high value for *X*_1_ and *X*_2_ (+1) or to its low value for *X*_3_ (−1). This choice was made in order to model less severe extrusion conditions.

## 3. Results and Discussion

### 3.1. Proximal Composition of Blends

Regarding the proximal composition of chickpea/rice flour blends (20/80, 30/70, and 40/60), significant changes were observed. Protein content increased from 9.84% (20/80) to 11.32% (40/60), reflecting the higher protein level in chickpea flour (15.74%) when compared to rice flour (~8.37%). Conversely, carbohydrates progressively decreased from 74.32% to 70.94%, due to chickpea flour’s lower carbohydrate content (60.82%) compared to rice flour (~77.69%). Ash content rose notably from 1.39% (20/80) to 1.71% (40/60), indicating higher mineral content in chickpea flour (2.66%) versus rice flour (~1.08%). The Total Energy Value (TEV) slightly decreased from 364.75 kcal/100 g (20/80) to 354.16 kcal/100 g (40/60), reflecting chickpea flour’s lower energy (322.38 kcal/100 g) relative to rice flour (~375.34 kcal/100 g).

### 3.2. Pasting Properties of Raw Ingredients

**Figure 1:**
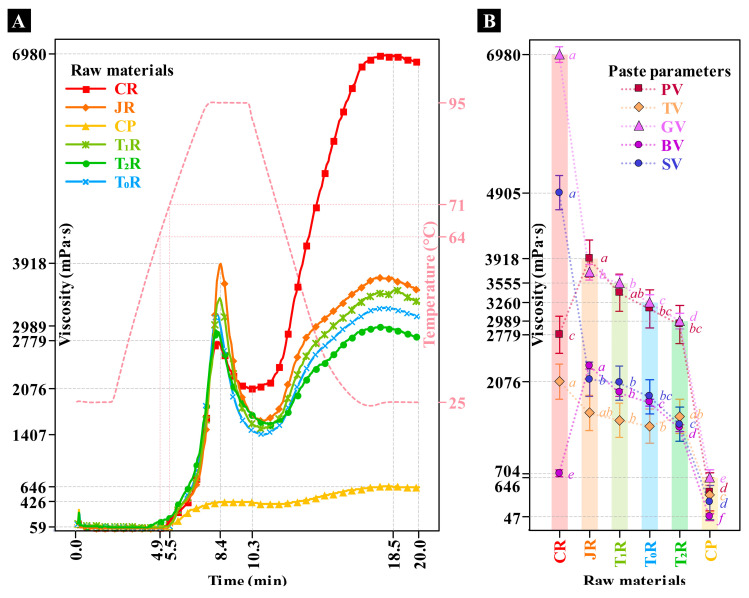
(**A**) RVA viscosity profiles of raw ingredients (Japanese rice: JR, chickpea whole particles: CP, and commercial rice flour: CR for comparison purposes) and feed materials for extrusion based on CP/JR proportions in dry basis (T_1_R: 20/80, T_2_R: 40/60, and T_0_R: 30/70). (**B**) Change in paste parameters: peak viscosity (*PV*), through viscosity (*TV*), gelation viscosity (*GV*), breakdown viscosity (*BV*), and setback viscosity (*SV*). Values with different lowercase letters, on a parameter, differ from each other according to the Tukey test.

Figure 1 presents the RVA curves of the raw ingredients and the feed materials for extrusion based on Japanese rice (JR), as chickpea (CP) whole particles were added.

Determining paste viscosity is essential to understand the effect of extrusion processing on the starch behavior, under cyclic hydrothermal conditions, achievable through rapid visco-analyzer (RVA). In Figure 1, the commercial rice (CR) exhibited the highest peak viscosity (*PV*, 6980 mPa·s), which is an indication of typical high-water absorption leading to great starch swelling, hence viscous paste formation. CP had significantly lower *PV* (426 mPa·s), suggesting limited water absorption and different thermal behavior. Mixtures of JR and CP showed intermediate values, with viscosity decreasing as chickpea content increased, which may be attributed in part to the dilution effect of starch, since it has other components such as protein rather starch granules. Samples T_1_R (20% CP), T_0_R (30% CP), and T_2_R (40% CP) clearly demonstrated the chickpea diluting effect on rice paste viscosity.

Figure 1B confirms these trends in specific viscosity parameters. CR again displayed significantly higher gelation viscosity (*GV*), setback viscosity (*SV*), and through viscosity (*TV*), indicating strong stability and gel-forming ability after cooling. In contrast, CP exhibited lower parameters, indicating reduced paste stability post-thermal treatment. Statistical analysis (Tukey) confirms significant differences between treatments, emphasizing the chickpea negative impact on viscoelastic properties, which was more pronounced at higher concentrations. Such findings are crucial for optimizing extruded product characteristics regarding texture and viscosity.

### 3.3. Physical Characterization in the Extrusion and Drying Processes

Table 1 presents the experimental design, the treatment code (the combination of actual levels for the factors), and the results of the feeder screw speed (*FSS*) and the semi-randomized extrusion order (*EO*) of treatments, designed considering the practical constraints of adjusting barrel temperature rapidly in the final heating zone. This approach allowed isolated assessment of input variables such as feed moisture and feeder screw speed without immediate interference from temperature variations. Table 1 outlines these parameters and their controlled variations, ensuring comparability and minimizing external influences among treatments. To achieve a feed mass flow rate of 4 kg/h, it was necessary to change *FSS* from 8 to 19 rpm due to differences in bulk density. Feed materials with lower bulk densities requires higher *FSS* to deliver the set mass flow rate. From Table 1, it can be inferred that T3, T4, T7, and T8 had the lowest bulk densities, which were significantly reduced by feed moisture and, to a lesser extent, by chickpea addition. Wet particles form clumps and increase in volume, which decreases interparticle density.

**Figure 2:**
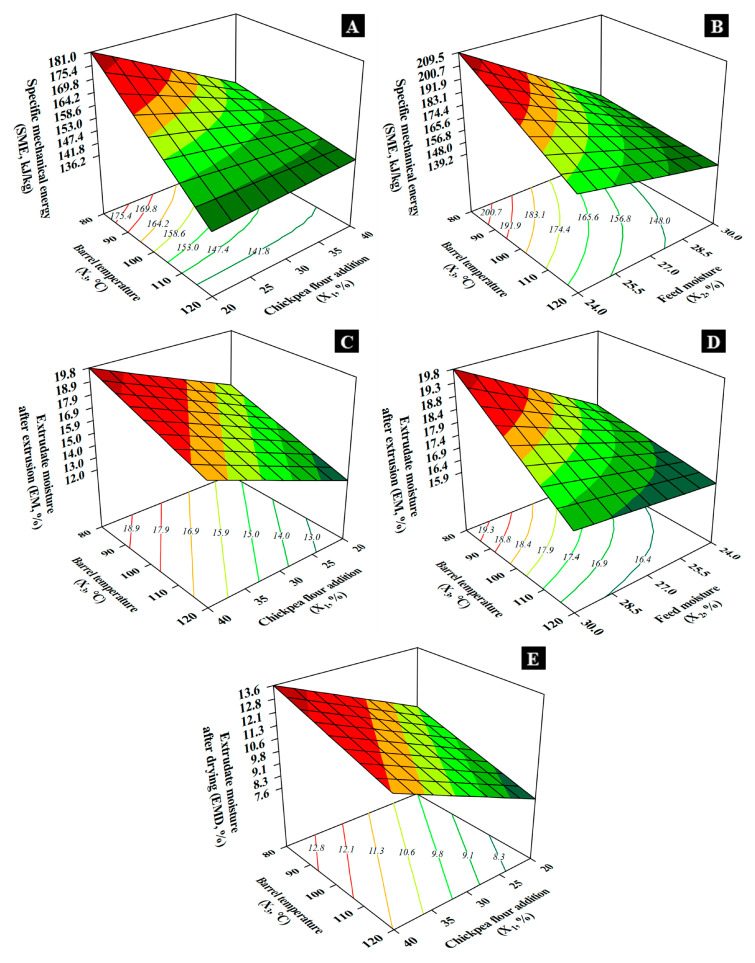
Effect of chickpea flour addition, feed moisture, and barrel temperature on (**A**,**B**) specific mechanical energy input during extrusion cooking; (**C**,**D**) extrudate moisture after extrusion; and (**E**) extrudate moisture after drying.

Chickpea addition (*X*_1_), feed moisture (*X*_2_), and barrel temperature (*X*_3_) inversely influenced specific mechanical energy (*SME*). *X*_1_ and *X*_2_ significantly impacted *SME*, both at low levels, and this impact was stronger at low temperatures (80 °C). *SME* ranged from 136.2 to 209.5 kJ/kg, peaking at 40% chickpea, 24% moisture, and 80 °C temperature (Figure 2A), and minimizing at 20% chickpea, 30% moisture, and 120 °C temperature (Figure 2B). Composition, particularly fiber, protein, and moisture levels, greatly influenced *SME* [19]. High temperatures reduced melt viscosity, varying with fiber content, moisture, and heating levels [20]. At 80 °C, elevated viscosity due to fibers and water reduced mechanical energy applied [21].

The final moisture of the extrudates before drying (*EM*) and after drying (*EMD*) is a function of the feed material moisture. In addition, moisture retention increased with chickpea addition and decreased with high cooking temperatures. For instance, at *X*_2_ = 30%, T4 (*X*_1_–*X*_3_: 40%–80 °C) presented the highest *EM* (19.08%) and *EMD* (14.22%), reflecting greater water retention capacity due to the fiber and protein contents contributed by the chickpea component. On the other hand, T7 (*X*_1_–*X*_3_: 20%–120 °C) showed the lowest *EM* (11.29%) and *EMD* (5.77%). The effect of *X*_3_ on moisture retention is due to more or less vaporization heat at high or low *X*_3_ level, respectively. Relating it to the *SME*, as more water retention by fiber and protein contents and less cooking temperature, the viscosity of the molten material increases, and consequently more mechanical energy is delivered by viscous dissipation [13], as compared the *SME* of T4 (161.40 kJ/kg) and T7 (138.37 kJ/kg).

*X*_1_ and *X*_2_ directly affected *EM*, particularly at low temperatures (80 °C). *X*_3_ inversely affected *EM* at high moisture (30%). *EM* ranged from 12.0% at 20% chickpea, 30% moisture, 120 °C temperature (Figure 2C) to 19.8% at 40% chickpea, 30% moisture, 80 °C temperature (Figure 2D). Extrudate moisture depends on water retention by fiber and protein content and initial moisture. Lower temperature favored moisture retention by reducing vaporization, while high temperature increased vaporization, lowering moisture content [6].

*X*_1_ directly influenced moisture content after drying (*EMD*), whereas *X*_3_ inversely affected it. *EMD* ranged from 7.6% at 20% chickpea, 120 °C temperature to 13.6% at 40% chickpea, 80 °C temperature (Figure 2E). Higher fiber and protein contents at lower temperatures increased moisture retention, demanding more drying energy due to density and water-retaining capacity [21].

### 3.4. Physical Characterization of Extruded Flours

Table 1 presents the physical properties of the EFs. The hydrothermal properties (solubility and hydration) of the particles suspended in water were strongly influenced by all the studied factors. Although the instrumental color parameters were not statistically affected by the studied factors, numerical differences were observed, as well as differences in the visual appearance of the extrudates.

Water solubility index (*WSI*) reflects the degree of molecular degradation, especially of starch. Higher chickpea inclusion increased *WSI* values, especially at low *SME*. For example, T8 (40% chickpea) showed the highest *WSI* (4.31 g ss/100 g db), while T4, also with 40% chickpea, had the lowest (1.85 g ss/100 g db), likely due to higher mechanical stress (*SME* = 161.40 kJ/kg) reducing matrix integrity. In contrast, treatments with 20% chickpea and higher *SME*, like T1, had moderate *WSI* (2.32 g ss/100 g db). *WSI* changes suggest that increased mechanical energy enhances molecular breakdown, solubilizing more solids into water.

**Figure 3:**
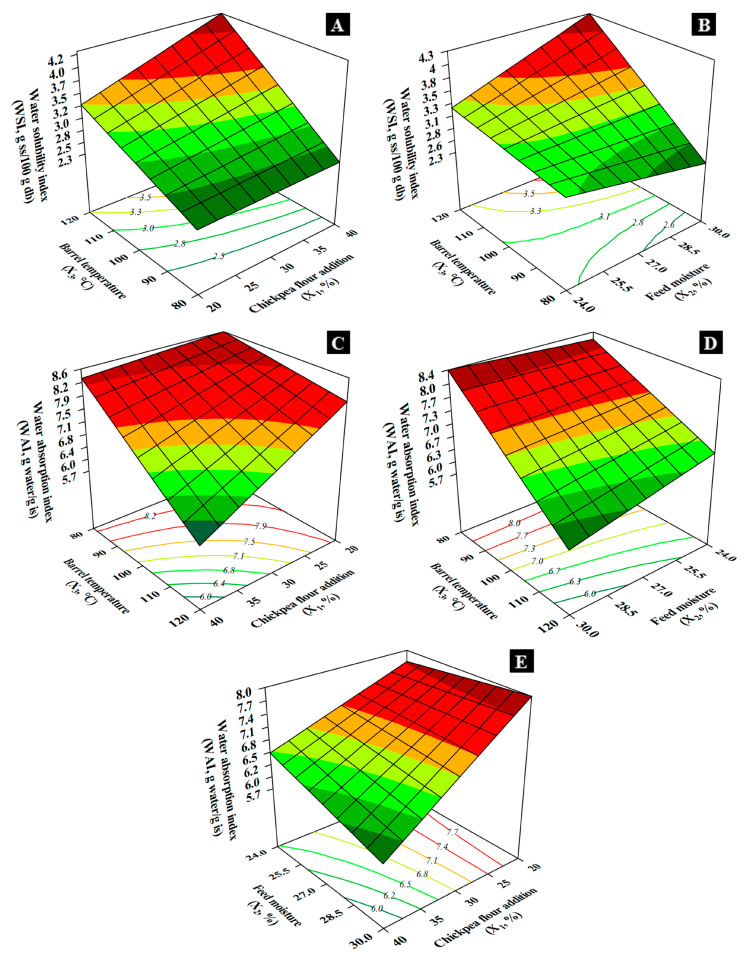
Effect of chickpea flour addition, feed moisture, and barrel temperature on (**A**,**B**) water solubility index and (**C**–**E**) water absorption index.

*X*_3_ significantly impacted *WSI* of the EFs, particularly at higher moisture levels (30%). *X*_1_ influenced *WSI* significantly at high temperatures (120 °C). *X*_2_ had opposite effects depending on temperature, being direct at high temperatures and inverse at low temperatures (80 °C). *WSI* ranged from 2.3 to 4.3 g ss/100 g db, reaching a maximum at 40% chickpea, 30% moisture, 120 °C temperature (Figure 3A) and a minimum at 20% chickpea, 30% moisture, 80 °C temperature (Figure 3B). Solubilization depended on extrusion severity, particularly thermal energy, causing starch breakdown and protein denaturation. Lower moisture increased shear force, enhancing macromolecule breakdown at 80 °C and 24% moisture [6].

Water absorption index (*WAI*) indicates the capacity of the material to retain water, influenced by starch gelatinization and fiber content. Lower *SME* values, as seen in T8 (*SME* = 141.04 kJ/kg), were associated with lower *WAI* (5.49 g water/g is), while higher *SME* values like in T1 (*SME* = 254.39 kJ/kg) resulted in higher *WAI* (7.67 g water/g is). Additionally, blends with lower chickpea content (T1, T3, T5) generally had higher *WAI*, likely due to the predominance of rice starch, which is more prone to shear degradation than pulse components. *WAI* changes indicate that shear can break structures but also partially expose water-binding sites.

*WAI* ranged from 5.7 to 8.6 g water/g is (Figure 3C), influenced by chickpea addition, moisture content, and temperature. Lowest *WAI* occurred at 40% chickpea, 30% moisture, 120 °C temperature (Figure 3D); highest *WAI* occurred at 20% chickpea, 30% moisture, 80 °C temperature (Figure 3E). Chickpea addition inversely affected *WAI* by introducing insoluble fibers, leading to reduced water absorption. High moisture and temperature exposed hydrophobic protein residues, further decreasing *WAI* [20].

Color attributes were influenced numerically but not statistically by chickpea addition and barrel temperature (Table 1). Chickpea addition led to lower L* values (darker appearance), as seen in T6 (L* = 87.51), compared to rice-dominant blends such as T1 (L* = 90.01). This darkening may result from Maillard reactions involving chickpea proteins and sugars, especially under high-temperature conditions. In terms of redness (a*), most treatments had low to slightly negative values, indicating little red pigmentation. However, T3 (a* = 1.77) and T6 (a* = 1.05) showed higher red tones, possibly due to more intense non-enzymatic browning at higher temperatures. The b* parameter, indicating yellow tones, increased with chickpea concentration and was generally higher in treatments with 40% chickpea (e.g., T2: b* = 19.04; T6: b* = 24.22; T8: b* = 20.02), reflecting the natural yellow pigments of chickpea and their concentration due to dehydration and cooking. Rice-dominant blends (T1, T5) showed lower b* values (~15–17).

### 3.5. Chemical Composition of Extruded Flours

The nutritional composition of the EFs (Table 1) was significantly affected by all the studied factors. Chickpea flour addition contributed to an increase in protein, lipid, ash, and dietary fiber content due to its inherently richer nutritional profile compared to rice flour.

Protein content (*PRO*) increased proportionally with chickpea addition, as seen in treatments with 40% chickpea (e.g., T2, T4, T6, and T8), where protein values consistently surpassed 11.2%. Conversely, formulations with 20% chickpea maintained lower protein levels (~9.8%). However, extrusion parameters also modulated protein content retention. For instance, despite having 40% chickpea, T6 exhibited slightly lower protein (11.29%) than T8 (11.40%), which could be attributed to different specific mechanical energy (*SME*) values (163.80 vs. 141.04 kJ/kg). Lower *SME* may preserve more protein integrity due to reduced mechanical degradation.

Carbohydrate content (*CHO*) tended to decrease with higher chickpea inclusion, as expected. Yet, anomalies such as T2 (75.01%) showing higher *CHO* than T1 (74.32%) suggest that *SME* and moisture content influenced starch degradation and solubility. Lower *SME* in T2 (200.75 kJ/kg) versus T1 (254.39 kJ/kg) may have contributed to less starch breakdown and solubilization, retaining more measurable carbohydrates.

Lipid content (*LIP*) increased in proportion to chickpea addition, with 40% chickpea blends reaching up to 2.92% (T2). However, T6 (2.77%) and T8 (2.81%) show slight lipid losses, likely due to higher barrel temperature (120 °C), which promotes lipid oxidation or evaporation. Additionally, lower *SME* (e.g., 138.37 kJ/kg in T7) was associated with better lipid retention, as excessive mechanical stress can disrupt oil bodies in chickpea.

Ash content (*ASH*) closely followed chickpea inclusion levels, with treatments at 40% consistently presenting values around 1.69–1.73%, reflecting the mineral-rich profile of legumes. Minor variations are observable across treatments, possibly due to mineral leaching or complexation during high-temperature processing.

Dietary fiber (*FIB*) also rose with chickpea addition. The highest values were found in 40% chickpea blends, especially T8 (5.82%) and T2 (5.81%). These results demonstrate that despite intense thermal and mechanical processing, a significant portion of insoluble dietary fiber is preserved. Notably, T8, processed at high temperature (120 °C), maintained its fiber integrity, possibly due to lower *SME*, which reduced mechanical disruption.

Finally, treatments with intermediate chickpea levels (30%), such as T9 to T12, showed stable nutritional patterns. T11 (with the lowest *SME*: 157.39 kJ/kg) resulted in slightly higher fiber values than T9 and T10, suggesting that moderate mechanical energy favors nutrient preservation.

### 3.6. Visual Characteristics of the Extruded and Noodles Produced

**Figure 4:**
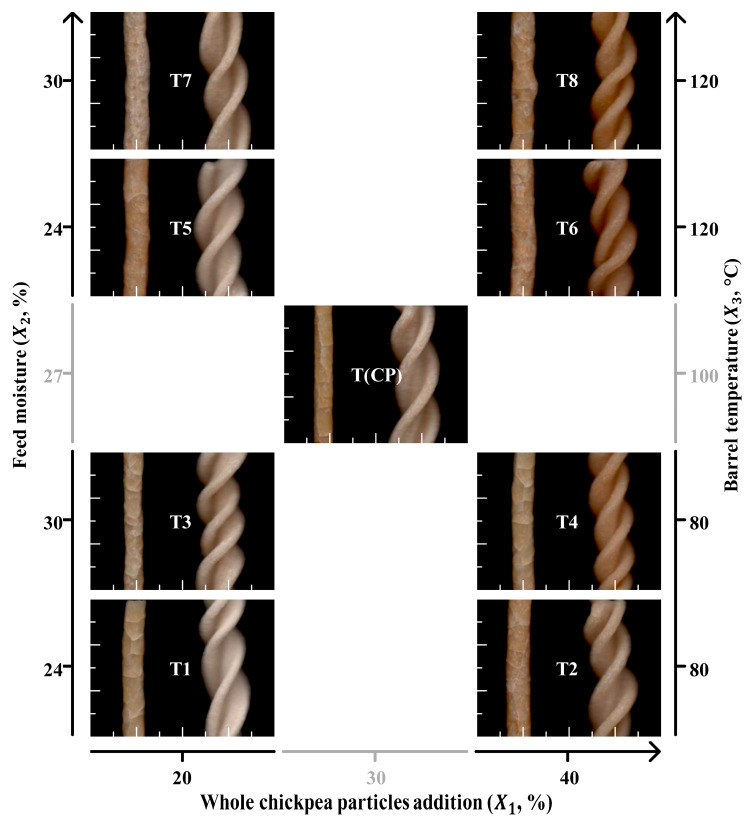
Scanned images of dried extrudates produced by extrusion cooking and their corresponding noodles shaped by cold extrusion. Image dimension: 4 cm × 3 cm. Treatment codes according to the experimental design: factorial points (T1-T8) and central points (T(CP)).

Figure 4 shows the expansion of the extrudates after drying and the visual appearance of the surface and shape of the extrudates and the corresponding dry noodles prepared with the EFs. The extrudate expansion was greater when the feed material with 20% chickpea addition was moistened to 24% and processed at 80% barrel temperature (T1 vs. T3). The effect of feed moisture on the extrudate expansion was less in the materials with 40% chickpea addition (T2 vs. T4) and it was virtually unaffected by chickpea addition and feed moisture when the materials were processed at 120°C (T5-T8).

The color of both products was darker due to 40% chickpea addition (treatments T2, T4, T6, and T8), and further darkened due to the increase in barrel temperature (T6 and T8). Compared to the extrudates, the color of the noodles was lighter in T1, T3, and T5 and darker in T4, T6, and T8. These differences reflect the interaction effect between chickpea addition and barrel temperature on the color of final products. Water retention in the dry extrudates (and consequently, in the EFs) affected the color of the dry noodles. EFs with 30% feed moisture, which have higher *EMD* (Table 1), produced darker noodles compared to extruded flours with 24% feed moisture.

The darker appearance of the products is likely associated with the pigments in the chickpea flour. Pigment retention in extrudates increases with high feed moisture and low barrel temperature. Furthermore, the starches and proteins present in feed materials (especially those with 40% chickpea addition) are likely to form reducing sugars and amino acids when modified during the extrusion process. Under moderate moisture (30% feed moisture) and high temperatures (120°C barrel temperature), these compounds are conducive to Maillard reactions that lead to changes in the aroma and color of extrudates [20,22].

It is likely that the chickpea-derived pigments like polyphenols and carotenoids reac with starches and proteins during extrusion. The higher protein content in chickpea improved noodle texture and structural integrity. The starch–protein and protein–lipid complexes formed in the noodle, enhanced hydration, elasticity, and firmness [23].

The extrusion process promotes starch–protein–lipid network formation, in particular the formation of hydrogen bonds between amylose and amylopectin, which significantly affects hydration, texture, and nutritional properties, in the latter case by influencing digestibility and nutrient bioavailability [24]. Thus, moisture, temperature, and ingredient proportions are critical in defining gluten-free noodle’s sensory, nutritional, and functional attributes, essential to optimize noodle products with enhanced sensory appeal and consumer acceptance [25].

### 3.7. Noodles Characterization

After determining the optimal cooking time (*OCT*) of each treatment (four intact pieces of dry noodles of ~5 cm), cooking tests and texture parameters were evaluated in the cooked noodles, which are summarized in Table 2.

#### 3.7.1. Cooking Tests

Cooking tests are essential for evaluating noodle quality. *OCT* varied between 9 and 11 min; T2 required the longest time (11 min), attributed to higher chickpea content requiring extended hydration and gelatinization. Most treatments cooked in 9–10 min indicated effective starch conversion caused by extrusion, by reducing cooking duration [26].

**Figure 5:**
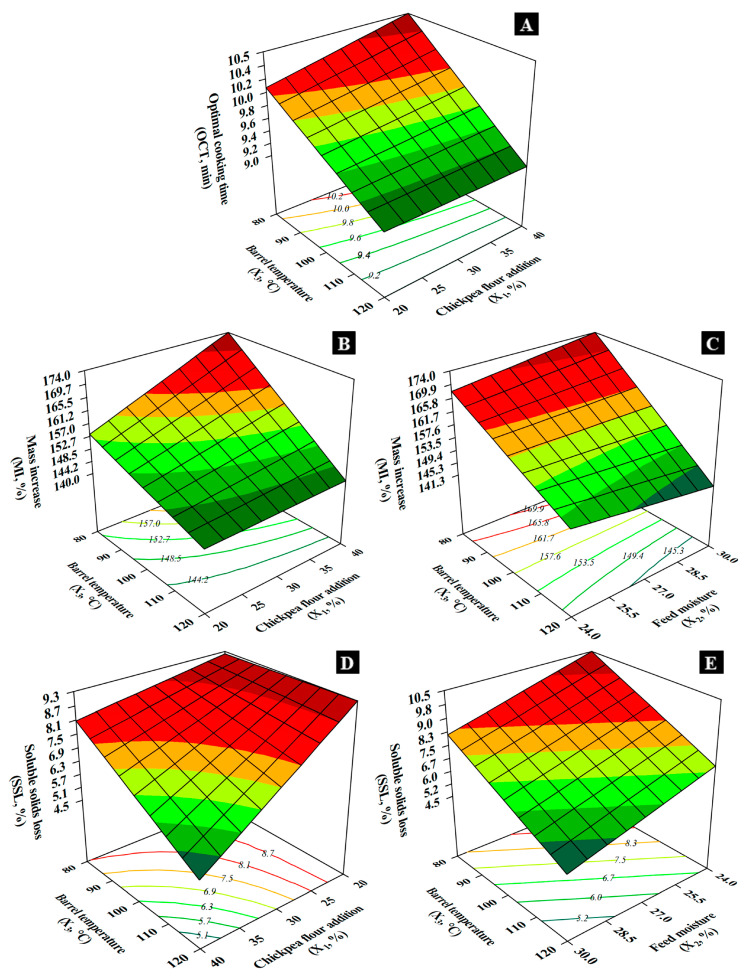
Effect of chickpea flour addition, feed moisture, and barrel temperature on (**A**) optimal cooking time; (**B**,**C**) mass increase; and (**D**,**E**) soluble solids loss.

Figure 5A shows the *OCT* of the noodles, which was slightly influenced by *X*_1_ in direct proportion only at low temperatures (80 °C), whereas *X*_3_ strongly and inversely impacted *OCT*. Longer values of *OCT* were observed at 80 °C, and shorter values at 120 °C, necessary to eliminate the opaque core of the noodle [12].

Mass increase (*MI*) and volume increase (*VI*) reflect water absorption capacity. T4 (172.50%) and T9 (158.76%) showed the highest *MI*, while T7 had the lowest (139.37%). Higher *MI* suggests greater water absorption due to hydrophilic components (starch, fibers (hemicellulose)). *VI* was highest in T4 (205.04%) and lowest in T7 (147.07%), indicating superior hydration and expansion for T4. *X*_3_ directly influenced noodle mass increase (*MI*), particularly at higher chickpea levels. *X*_2_ directly affected *MI* notably at high temperatures (120 °C) but inversely at lower temperatures (80 °C). *MI* ranged from 140.0% at 20% chickpea, 30% moisture, 120 °C temperature (Figure 5B) to 174.0% at 40% chickpea, 30% moisture, 80 °C temperature (Figure 5C).

Soluble solids loss (*SSL*), indicative of noodle integrity, was highest in T2 (10.17%) and T3 (9.11%), signifying weaker starch–protein interactions. T8 had the lowest *SSL* (4.12%), reflecting better structure retention during cooking [27]. *X*_1_ significantly affected *SSL* in the cooked noodles, notably at high temperatures (120 °C), the effect being less at 80 °C (Figure 5D). *X*_2_ and *X*_3_ inversely influenced *SSL* (Figure 5E), which ranged from 4.5% (40% chickpea, 30% moisture, 120 °C temperature) to 10.5% (40% chickpea, 24% moisture, 80 °C temperature).

#### 3.7.2. Texture Profile Analysis

The texture profile analysis parameters reveal noodle quality and, indirectly, consumer sensory preferences, especially regarding the perception of ‘al dente’ texture, as reported in previous studies. As hardness (*HA*) is a measure of compression force, T1 (29.17 N) and T7 (20.15 N) exhibited the highest hardness, indicating firmer texture. T9 (4.12 N) and T5 (4.56 N) had the lowest hardness, indicating softer textures. *HA* differences between treatments is related to starch conversion and protein denaturation, probably influenced by chickpea proteins [19].

As elasticity (*EL*) would be defined as the noodle’s ability to recover shape after deformation, it was higher in T4 (2.30) and T9 (2.22) and lower in T2 (0.88). High *EL* relates to effective amylose, amylopectin, and protein cross-linking, were essential for keeping the fusilli shape post-cooking [12].

Cohesiveness (*CO*), defined as the internal binding force, ranged between 0.84 (T8) and 0.71 (T10 and T12). Higher *CO* indicates stronger molecular interactions, enhancing noodle structural integrity. Gumminess (*GO*) and chewiness (*CW*) reflect firmness and chewing resistance. Highest *GO* was recorded in T1 (21.09 N) and T7 (15.13 N), correlating with *HA*, while T9 (3.02 N) and T10 (3.15 N) had the lowest values. *CW* was highest in T1 (19.70 N) and T3 (14.09 N) and lowest in T10 (3.02 N) and T9 (3.11 N), confirming a soft bite [8].

Concerning the correlation between cooking tests and texture parameters, it was observed that higher mass and volume increased as T4 and T9 typically displayed softer textures, suggesting excessive hydration that compromised noodle firmness. *SSL* inversely correlated with *CO* as shown in treatments T2 and T12, since they presented high *SSL* and exhibited lower *CO*, probably due to starch leaching that weakened their structural integrity. *HA* positively correlated with *CW*, indicating that a firmer noodle requires greater chewing effort [20].

The influence of the studied factors was observed among treatments, higher moisture during extrusion enhances hydration but may reduce structural integrity [22]. Lower hardness in T9 and T5 indicates excessive starch conversion, resulting in softer texture. Higher temperatures strengthen starch–protein interactions, which may have caused starch breakdown, thus affecting firmness [12]. Treatments produced at elevated temperatures (e.g., T4, T8) exhibited improved elasticity and cohesiveness in detriment of increasing gumminess when starch breakdown was excessive during extrusion cooking. As for ingredient formulation, chickpea-to-rice flour ratios influenced protein–starch matrix interactions. It has been reported that chickpea proteins increased elasticity and cohesiveness, but excessive amounts raised soluble solids loss, negatively affecting structural integrity [26].

Optimal texture in gluten-free noodles thus requires balancing hydration, processing parameters, and ingredient composition. Treatments T1, T3, and T7 displayed firmer textures, indicating better protein–starch interactions and adequate level of starch gelatinization. Softer textures observed in T5 and T9 could affect consumer preferences. Moderated hydration, controlled cooking, and optimized protein–starch ratios are critical for achieving high quality, desirable noodle texture and stability [28].

**Figure 6:**
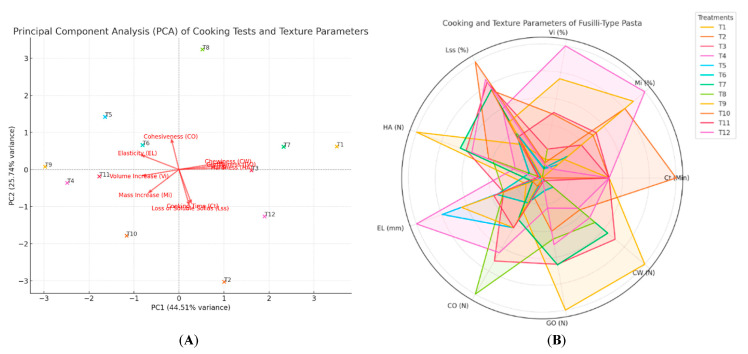
(**A**) Principal component analysis (PCA) plot and (**B**) radar chart, both based on the cooking and texture parameters; *OCT*: optimum cooking time, *MI*: mass increase, *VI*: volume increase, *SSL*: soluble solids loss. *HA*: hardness, *EL*: elasticity, *CO*: cohesiveness, *GO*: gumminess, *CW*: chewiness.

Treatment variations in cooking tests and texture parameters were visualized as principal component analysis (PCA, Figure 6A) and radar charts (Figure 6B), which illustrate the impacts of cooking and texture parameters, emphasizing the intricate balance required between formulations and processing to achieve desirable gluten-free noodle characteristics [20].

These findings demonstrate how optimal gluten-free noodle production involves carefully balancing chickpea addition, moisture levels, and extrusion temperatures to achieve desired cooking and textural properties.

## 4. Conclusions

The present study aimed to develop gluten-free fusilli-type noodles using extruded Japanese rice and chickpea flours and evaluate their cooking properties, texture parameters, and overall structural characteristics. The results demonstrated that formulation composition, processing conditions (moisture content and extrusion temperature), and ingredient interactions significantly influenced the quality attributes of the final product. Increasing the proportion of chickpea flour resulted in darker noodles, likely due to the natural pigments present in chickpeas, which were further intensified by the higher moisture and heat energy during extrusion. The optimal cooking time varied slightly among formulations, ranging from 9 to 11 min. Higher mass and volume increases were observed in treatments with greater chickpea content, likely due to the higher water absorption capacity of legume-derived proteins and starches. However, increased water content during extrusion (30%) led to greater solid losses (L_ss_), indicating that some formulations exhibited higher starch solubilization and potential loss of structural integrity. An optimal formulation would involve a balanced proportion of rice and chickpea flour, maintaining an extrusion moisture level around 25% to ensure adequate hydration while minimizing excessive solid loss. A two-component mixture composed of 40% chickpea flour and 60% Japanese rice, moistened to 30% and processed by extrusion at 80 °C, ensured processing efficiency. These findings highlight the importance of fine-tuning extrusion parameters and formulation compositions to achieve high-quality gluten-free noodles. The study contributes to the growing field of alternative noodle development by demonstrating how ingredient interactions, moisture content, and processing conditions can be manipulated to enhance gluten-free noodle performance with better nutritional quality. Future studies should focus on refining formulation strategies, assessing sensory acceptance, and evaluating long-term storage stability to ensure commercial viability.

## Figures and Tables

**Table 1:** Experimental design and properties evaluated in the extrusion-drying processes and in the extruded flours.

**Experimental**				**Treatment**	**Process Properties**							**Extruded Flour Properties**											
**Design**					**Extrusion**				**Drying**			**Hydration**			**Instrumental Color**				**Chemical Composition**				
*X*_1_	*X*_2_	*X*_3_		**Code**	*EO*	*FSS*	*SME*		*EM*	*EMD*		*WSI*	*WAI*		L*	a*	b*		*PRO*	*CHO*	*LIP*	*ASH*	*FIB*
%	%	°C		-	-	rpm	kJ/kg		%	%		% ^1^	g/g ^2^		-	-	-		%	%	%	%	%
20	24	80		T1	4°	8	254.39		13.68	8.71		2.32	7.67		90.01	0.05	15.67		9.84	74.32	1.81	1.39	3.56
40	24	80		T2	11°	10	200.75		16.72	12.51		3.00	7.96		89.27	−0.19	19.04		11.38	75.01	2.92	1.71	5.81
20	30	80		T3	5°	17	172.26		15.27	10.02		2.40	8.41		87.20	1.77	24.55		9.85	74.29	1.79	1.41	3.56
40	30	80		T4	10°	19	161.40		19.08	14.22		1.85	8.72		90.44	0.15	16.65		11.29	71.01	2.85	1.72	5.77
20	24	120		T5	2°	8	160.33		13.53	8.97		2.23	7.51		90.02	−0.03	17.06		9.81	74.38	1.84	1.42	3.49
40	24	120		T6	7°	10	163.80		15.14	10.60		2.85	6.89		87.51	1.05	24.22		11.29	71.24	2.77	1.69	5.79
20	30	120		T7	1°	17	138.37		11.29	5.77		2.90	8.33		90.55	0.14	15.90		9.79	74.22	1.82	1.36	3.48
40	30	120		T8	8°	19	141.04		16.45	11.03		4.31	5.49		88.93	0.13	20.02		11.40	72.11	2.81	1.73	5.82
30	27	100		T9	3°	14	171.32		16.37	10.33		3.09	7.13		90.13	−0.15	17.11		10.52	6.91	2.32	1.55	4.66
30	27	100		T10	6°	14	168.81		15.69	11.03		3.34	7.26		89.12	−0.17	18.92		10.55	72.64	2.35	1.53	4.59
30	27	100		T11	9°	14	157.39		16.95	12.00		3.48	7.67		89.05	−0.25	18.67		10.50	71.98	2.31	1.56	4.69
30	27	100		T12	12°	14	174.76		16.47	11.88		3.04	7.37		90.14	−0.17	17.29		10.51	72.12	2.41	1.54	4.68

*X*_1_: addition of chickpea flour; *X*_2_: feed moisture; *X*_3_: barrel temperature in the third zone; *EO*: extrusion order; *FSS*: feeder screw speed; *SME*: specific mechanical energy; *EM:* extruded moisture; *EMD:* moisture of extrudates after drying; *WSI*: water solubility index (^1^ g ss/100 g db); *WAI*: water absorption index (^2^ g water/g is); L*: lightness; a*: red-green color; b*: yellow-blue color; *PRO*: protein; *CHO*: carbohydrates; *LIP*: lipids; *ASH*: ashes; *FIB*: dietary fiber.

**Table 2:** Average of the noodles cooking tests and texture parameters.

Treatment		Cooking Tests ^1^					Texture Parameters ^2^				
		*OCT*	*MI*	*VI*	*SSL*		*HA*	*EL*	*CO*	*GO*	*CW*
		min	%	%	%		N	-	-	N	N
T1		10	145.82	155.60	5.86		29.17	0.93	0.74	21.09	19.70
T2		11	165.93	154.12	10.17		14.04	0.88	0.73	10.17	9.54
T3		10	151.51	160.02	9.11		19.50	1.09	0.79	15.64	14.09
T4		10	172.50	205.04	7.72		9.21	2.30	0.80	7.12	9.21
T5		9	143.94	151.99	6.48		4.56	2.05	0.75	3.45	4.49
T6		9	147.36	152.40	6.53		6.51	2.02	0.73	5.52	5.20
T7		9	139.37	147.07	8.72		20.15	0.94	0.75	15.13	13.67
T8		9	138.51	160.44	4.12		14.11	1.07	0.84	11.47	11.19
T9		9	158.76	191.59	9.06		4.12	2.22	0.77	3.02	3.11
T10		10	155.36	175.28	8.68		5.70	0.95	0.71	3.15	3.02
T11		10	156.39	176.36	7.77		5.11	2.21	0.79	4.17	4.14
T12		10	152.02	152.55	9.25		18.52	0.89	0.71	12.08	10.04

^1^ Average of 3 replicates, ^2^ Average of 16 replicates. *OCT*: optimum cooking time, *MI*: mass increase, *VI*: volume increase, *SSL*: loss of soluble solids. *HA*: hardness, *EL*: elasticity, *CO*: cohesiveness, *GO*: gumminess, *CW*: chewiness.

## Data Availability

The original contributions presented in this study are included in the article. Further inquiries can be directed to the corresponding author(s).

## Supplementary Materials

The following are available online at https://www.mdpi.com/article/10.3390/foods14142524/s1, Figure S1: Schematic illustration of the process.
