# Supplementary material for: Innovative Gluten-Free Fusilli Noodle Formulation: Leveraging Extruded Japanese Rice and Chickpea Flours
Source: Foods. 2025 Jul 18;14(14):2524. doi: 10.3390/foods14142524 (PMC12294755; doi:10.3390/foods14142524)
Supplement: Supplementary file 1 [file foods-14-02524-s001.zip › foods-3726764-supplementary.pdf]

Supplementary Materials

A schematic illustration of the process is provided, highlighting a separation between pre-cooked flour and noodle productions:

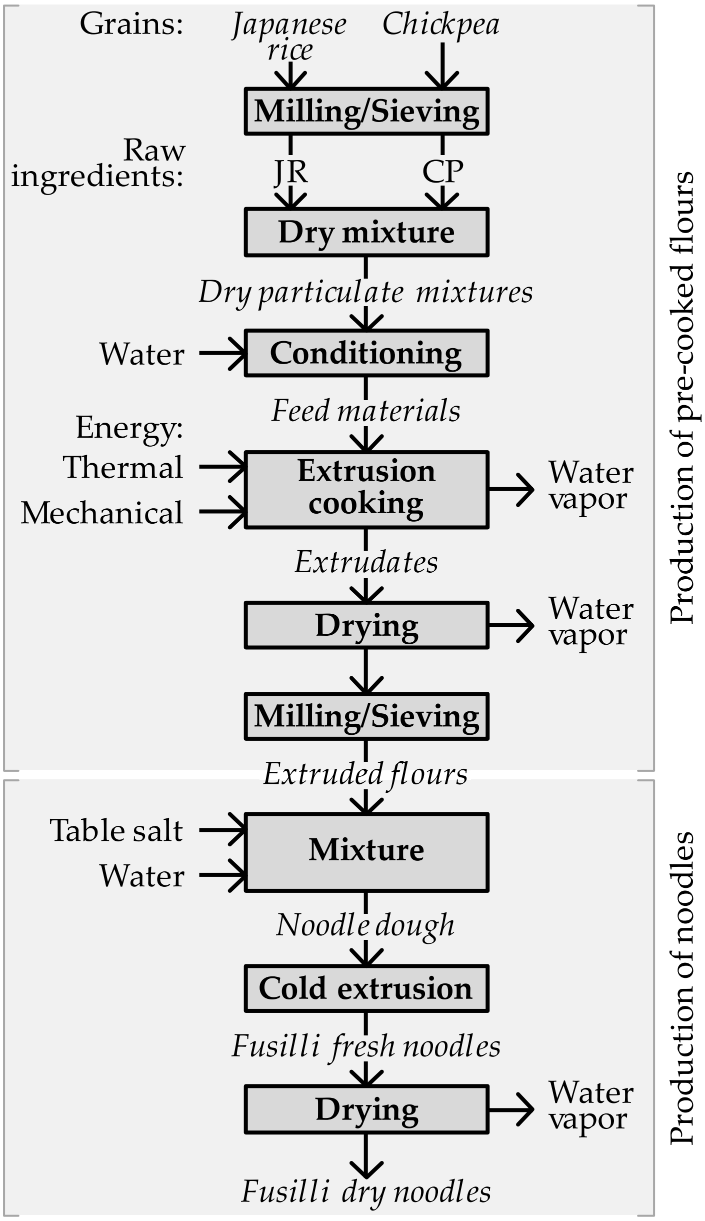

Figure S1: Schematic illustration of the process.
